# Retrospective study of pleural parasitic infestations: a practical diagnostic approach

**DOI:** 10.1186/s12879-019-4179-9

**Published:** 2019-07-04

**Authors:** Jinlin Wang, Weizhan Luo, Panxiao Shen, Jianxing He, Yunxiang Zeng

**Affiliations:** 1 Department of Respiratory Disease, The State Key Laboratory of Respiratory Disease, China Clinical Research Centre for Respiratory Disease, Guangzhou Institute of Respiratory Disease, First Affiliated Hospital of Guangzhou Medical University, Guangzhou, China; 2 Department of Cardiothoracic Surgery, The State Key Laboratory of Respiratory Disease, China Clinical Research Centre for Respiratory Disease, Guangzhou Institute of Respiratory Disease, First Affiliated Hospital of Guangzhou Medical University, 151 Yanjiang Road, Guangzhou, 510120 Guangdong Province China

**Keywords:** Pleural parasitic infestation, Pleural effusion, Eosinophilic pleural effusion

## Abstract

**Background:**

Pleural parasitic infestation (PPI) is a disease prevalent in certain parts of the world. It is frequently misdiagnosed due to its lack of standardized diagnostic criteria. The purpose of this study was to evaluate the clinical characteristics of PPI patients and develop a practical diagnostic approach for PPI.

**Methods:**

A retrospective study was conducted by reviewing the medical records of 11 patients with PPI. A practical diagnostic approach was proposed based on the unique laboratory findings.

**Results:**

All patients demonstrated respiratory symptoms, including shortness of breath, cough, fever, chest pain, excessive sputum and hemoptysis. Leukocytosis (> 10,000/μL) and eosinophilia (> 500/μL) of peripheral blood were present in 45.5 and 36.4% patients, respectively. The mean concentrations of pleural effusion lactate dehydrogenase (LDH), adenosine deaminase (ADA), protein and carcinoembryonic antigen (CEA) were 338.2 U/L (range, 61–667 U/L), 11.6 U/L (range, 0.1–28.2 U/L), 43.7 g/dL (range, 21.9–88.1 g/dL), and 1.84 mg/mL (range, 0.28–4.8 mg/mL), respectively. The mean percentage of eosinophils in the pleural effusion was 19.5% (10.5–41%). Blood test was positive for parasite-specific IgG antibody in 9 patients, including 4 for *Paragonimus westermani, 3 for Taenia solium, 1 for Clonorchis sinensis and 1 for Echinococcus granulosus.* Eggs of *Clonorchis sinensis* were detected in the stool of two patients. *Sparganum* was found in the pleural effusion of one patient. Respiratory symptoms and abnormal appearances in pulmonary radiographic examination were disappeared in all patients who received anti-parasitic treatment.

**Conclusions:**

In patients with unexplained pleural effusion, parasite-specific IgG antibody tests should be performed when pleural fluid testing shows eosinophilic pleural effusion. It is preferable to consider the diagnosis of PPI in clinical practice when serum parasite-specific IgG antibody test is positive.

## Background

Pleural parasitic infestation (PPI) is an extremely rare pleural disease caused by a variety of parasites, such as the lung fluke *Paragonimus westermani, Toxocara* spp*., Clonorchis sinenis, Spirometra* spp. and *Taenia solium,* etc. [1, 2]. Recently, more and more new PPI cases have been reported throughout the world [3–10]. PPI may only have pleural involvement, or other non-specific pulmonary manifestations [3, 11–13]. Previous studies have found that pleural involvement is more common than intrapulmonary parenchymal lesions in patients with pulmonary paragonimiasis [14, 15]. There are different pleural manifestations in patients with PPI, such as pleural thickening, pleural effusion, empyema or chylothorax. The clinical manifestations are non-specific and many patients are initially treated for tuberculosis or misdiagnosed as lung cancer [16]. Consumption of raw or undercooked fresh water crab, or crayfish infected with *Paragonimus* metacercariae is the main source of infection in human [3]. Trichomonad parasites probably enter the respiratory tract following aspiration of oropharyngeal secretions [10]. However, due to the expansion of worldwide food trading and increasing number of travelers and immigrants, the exposure history of the infection in each individual person is becoming more and more obscure.

Eosinophilic pleural effusion (EPE) defined by an eosinophil count of ≥10% in the pleural fluid accounts for 5 to 16% of exudative pleural effusions [17]. EPE occurs most commonly during conditions associated with the presence of blood or air in the pleural space, infections, and malignancy. As an important cause of EPEs, PPI is a curable condition [18, 19]. Among those that cause EPE, the most common parasites are *Paragonimus spp.* which is endemic in eastern and southeastern Asia. Other parasites associated with EPEs include *Spirometra* spp*.* [20], *Toxocara* spp [21], and *Echinococcosis granulosus* [22]. Current guidelines on the investigation of pleural effusions suggest the use of a diagnostic algorithm or a stepwise approach [23–26]. However, the presence of EPE is not taken into consideration by the current guidelines.

Although human PPI have been sporadically reported worldwide, only a few reports have analyzed the clinical features, diagnosis and treatment of paragonimiasis [3, 11–13]. Thus, it is challenging for physicians to make a definitive diagnosis. Delayed diagnosis or misdiagnoses causes increased morbidity and mortality. In the present report, we proposed a practical diagnostic approach after analyzing the medical records of 11 cases of PPI patients.

## Methods

Eleven PPI patients admitted to the First Affiliated Hospital of Guangzhou Medical University from January 2010 to January 2016 were retrospectively reviewed. The patients were admitted to our hospital due to respiratory symptoms and abnormal chest high-resolution computed tomography (HRCT) findings that revealed pleural effusion or pleural pulmonary involvement. Thoracocentesis and a pleural biopsy were performed in each case. The enzyme-linked immunosorbent assay (ELISA) test for parasite-specific IgG antibodies (Guangzhou Yikang Biotechnology Co. Ltd.) was performed on serum from all patients and on pleural effusion from 2 patients. The parasite-specific IgG antibodies included the IgG antibodies of *Taenia solium*, *Paragonimus westermani,* and *Spirometra* spp.*, Clonorchis sinensis, Toxoplasma gondii* and *Echinococcus granulosus*. Stool examinations for the detection of parasite eggs were performed in all patients.

### Diagnosis of PPI

A practical diagnostic approach for PPIs were proposed based on: [1] the pleural involvement and the feature of pleural effusion. [2] immunoserologic test result for a parasite-specific antibody, and/or on the detection of characteristic parasite eggs (in the pleural effusion, sputum, bronchial washing fluid, lung biopsy specimens or stool). Patients with presumptive diagnosis of PPIs were prescribed antiparasitic treatment and followed up over up to 15 months.

### Data analysis

A systematic review of the patients’ clinical data was performed, including symptoms, their exposure history of raw or undercooked freshwater crab or crayfish intake, laboratory test results, and other diagnostic procedures.

## Results

### Patients’ characteristics

The study population consisted of 11 patients (8 males and 3 females) with a median age of 51.1 years (range, 20 to 81 years). Their clinical characteristics were shown in Table 1. Respiratory symptoms were presented in most of the patients (9 of 11). The main complaints included shortness of breath (*n* = 4; 36.4%), cough (n = 4; 36.4%), fever (n = 4; 36.4%), chest pain (*n* = 3; 27.3%), excessive sputum (*n* = 2; 18.2%) and hemoptysis (*n* = 1; 9.1%). There were another 4 patients who had fever as a systemic reaction. The duration of complaints ranged from 2 days to more than 2 years. The patients were initially misdiagnosed as tuberculosis pleural effusion (TPE), idiopathic eosinophilic pleural effusion (IEPE), parapneumonic pleural effusion (PPE) or pulmonary embolism (PE). Only 4 patients (36.4%) had a consumption history of raw freshwater crab ingestion (patients No. 7 and 9), raw fish ingestion (patient No. 3) or raw wild boar meat ingestion (patient No. 10). No remarkable exposure history was found in the other 7 patients.

**Table 1:** Demographic characteristics of the 11 patients with parasitic pleural infestation

Patient No.	Chief complaint (duration)	Misdiagnosis	Exposure history	Peripheral blood		Pleural effusion				
				WBC (× 10 [9]/L)	Eos (×10 [9]/L)	LDH (U/L)	ADA (U/L)	Protein (g/dL)	CEA (ng/mL)	Eos (%)
1	Chest pain (6 mth); shortness of breath (2 mth)	PPE	Unknown	7.33	0.872	346	8.2	30.9	0.28	15.3
2	Chest pain (12 mth); shortness of breath (2 mth)	TPE	Unknown	4.61	0.337	125	6.9	88.1	0.67	10.5
3	Chest pain, shortness of breath (6 mth)	TPE	Raw fish ingestion	7.4	0.799	150	2.2	36.9	0.69	14.5
4	Cough, shortness of breath (1 mth)	TPE	Unknown	4.6	0.736	367	3.4	45.3	2.03	19.7
5	Fever (12 d)	PPE	Unknown	4.7	0.611	128	6.7	43.2	4.8	13.5
6	Fever (3 wk)	IEPE	Unknown	7.4	1.450	61	0.1	21.9	0.5	10.8
7	Cough, hemoptysis, fever (2 d)	PTE	Raw freshwater crab ingestion	12.7	0.340	587	19.1	43.1	1.74	30.2
8	Cough, shortness of breath (4 mth)	IEPE	Unknown	16.7	5.778	667	10.8	47.4	0.3	22.5
9	Cough, excessive sputum (24 mth); fever (1 mth)	IEPE	Raw freshwater crab ingestion	18.8	9.926	564	19	45	2.4	41
10	shortness of breath (6 mth);, cough (1 mth)	PPE	Raw wild boar meat ingestion	6.8	0.380	457	23.1	42.1	4.2	14.0
11	Fever, chest pain (2 wk)	PE	Unknown	11.8	0.472	268	28.2	36.3	2.7	22.1

Abbreviations*: ADA*, Adenosine deaminase; *CEA*, Carcinoembryonic antigen; *d*, day; *Eos*, Eosinophils; *IEPE*, Idiopathic eosinophilic pleural effusion; *LDH*, Lactate dehydrogenase; *mth*, months; *PE*, Pulmonary embolism; *PPE*, Parapneumonic pleural effusion; *TPE*, Tuberculosis pleural effusion; *WBC*, White blood cell; *wk*., weeks

### Laboratory findings and radiologic features

**Fig. 1:**
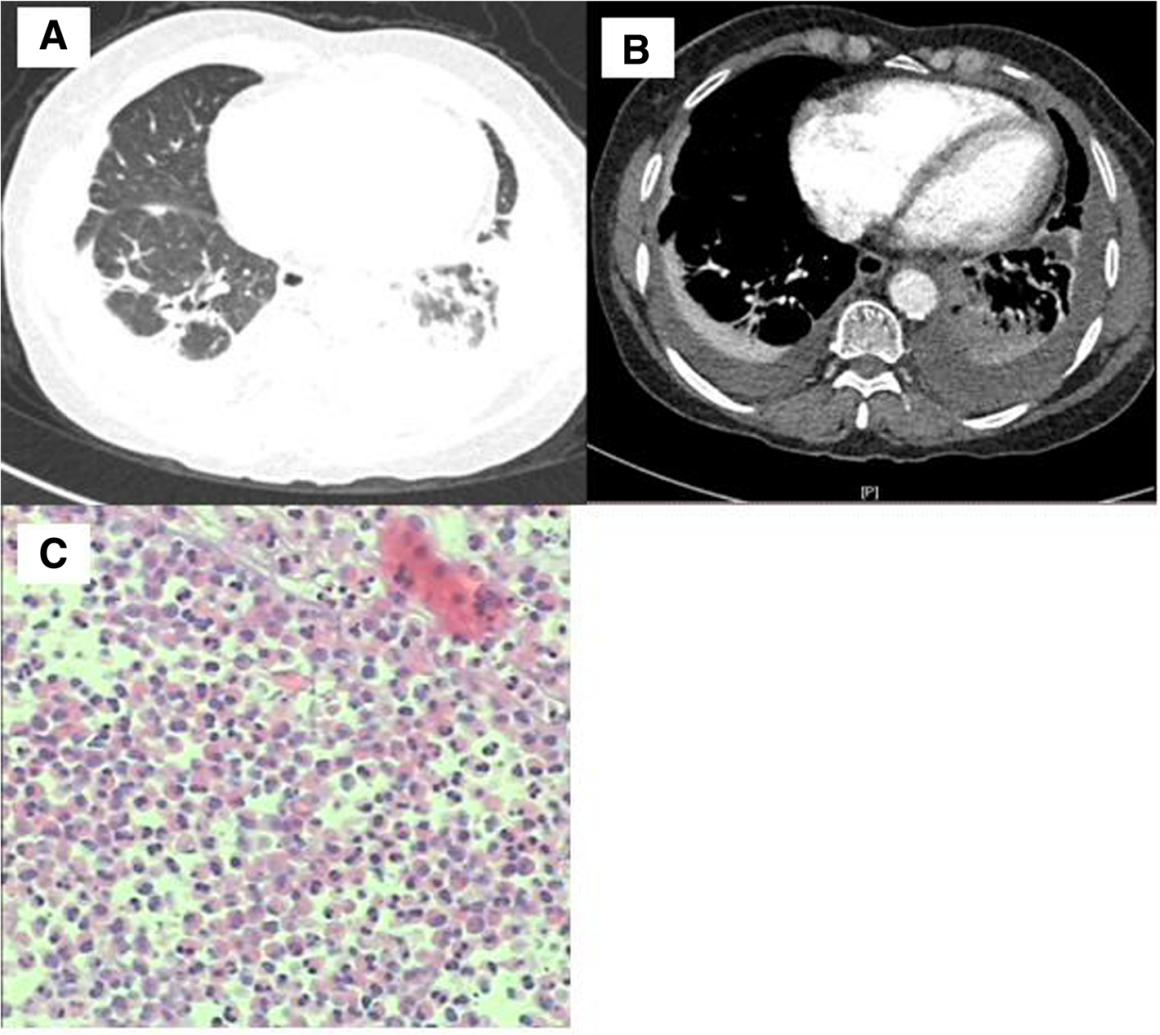
A woman of PPI with pulmonary cysticercosis. A chest CT scan of the patient showed bilateral pleural effusion and consolidation in the lower right lung (**a**, **b**). Multiple eosinophlic infiltration with no neoplastic cells showed in histopathological examination of a pleural sample (C)

Patients with PPI presented with a variety of radiographic features. Pleural effusion was observed in all patients. Unilateral pleural effusion was seen in 8 patients and bilateral pleural effusion was found in the remaining 3 patients. As shown in Fig. 1 a and b, the imaging findings included large area of consolidation, nodular/mass as well as enlargement of the mediastinal lymph node. Even though it is not specific to PPI, these intrapulmonary involvements were seen in a majority of patients (7/11; 63.6%).

**Fig. 2:**
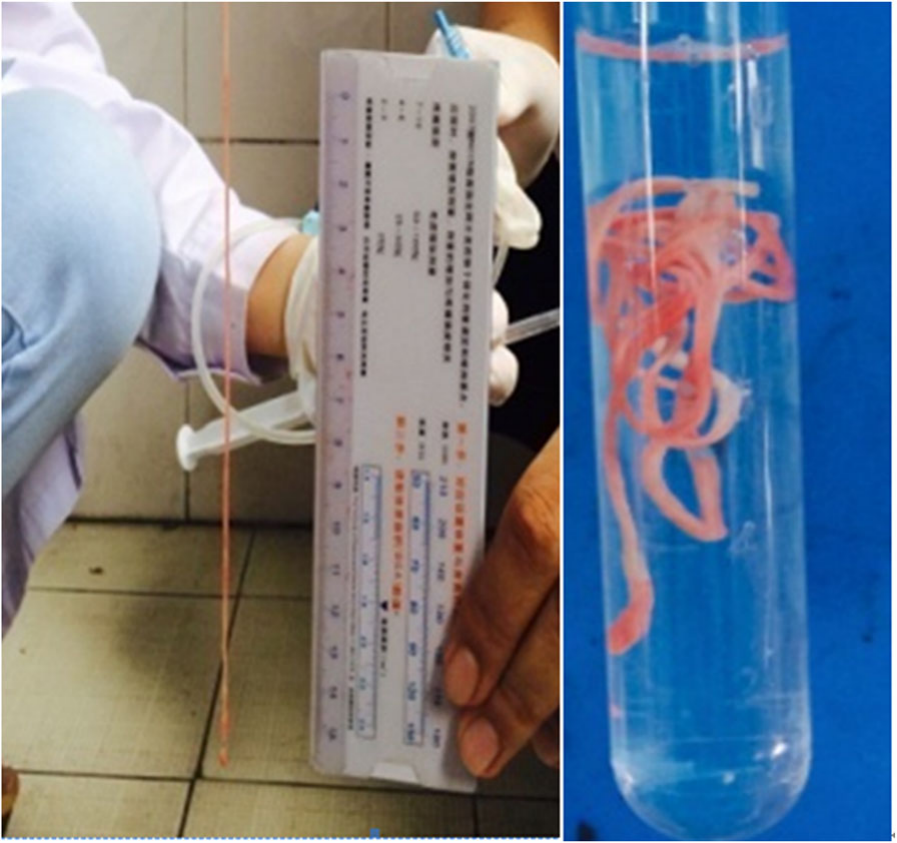
A of PPI with pulmonary sparganosis, which was found and confirmed in pleural effusion

Peripheral blood cell analysis was performed in all patients. Eosinophilia of peripheral blood (> 500/μL) was present in 5 patients (45.5%) while it was within the normal range in the other 6 patients. Leukocytosis (> 10,000/μL) was detected in 4 patients (36.4%). Serum IgE levels were tested in 8 patients and only 3 of them had elevated serum IgE (37.5%). IgG antibody of *Paragonimus westermani* were found in the serum of 4 patients. *Taenia solium* IgG antibody was found in 3 patients. *Echinococcus granulosus* and *Clonorchis sinensis* specific antibody was respectively detected in the blood samples of 2 other individuals. *Clonorchis sinensis* eggs were present in the stool of 2 patients. *Sparganum* was found and confirmed in the pleural effusion of patient No. 3 (Fig. 2).

### Examination of pleural effusion and the pathology of pleura

Thoracocentesis and pleura biopsy were performed in all patients, and pleural effusion data were available for analysis. EPE was detected in all patients. The mean percentage of eosinophils in pleural effusions was 19.5% (range, 10.5–41%). Other pleural effusion parameters were also measured and the data were shown in Table 1. The mean concentrations of lactate dehydrogenase (LDH), adenosine deaminase (ADA), protein, and carcinoembryonic antigen (CEA) were: 338.2 U/L (range, 61–667 U/L), 11.6 U/L (range, 0.1–28.2 U/L), 43.7 g/dL (range, 21.9–88.1 g/dL), and 1.84 mg/mL (range, 0.28–4.8 mg/mL), respectively. Pleural samples were acquired by combining ultrasound-guided cutting needle biopsy and standard pleural biopsy [27]. In 8 of 11 patients, eosinophilic infiltration was found but there was no evidence of tuberculosis or malignancy in any patient. Parasite eggs were not seen in any of the pleural biopsies. Histopathological examination shown in Fig. 1C demonstrated multiple eosinophilic infiltrations in the pleural sample of patient No. 2. Lung biopsy was performed in 6 patients and eosinophilic infiltration was found in 50% of these patients (Table 2).

**Table 2:** Diagnosis and treatment of the 11 patients with PPI

Patient No.	Parasite eggs (stool)/parasite)	Parasite antibody	Eos of PL/pleural biopsy/ lung biopsy	Treatment	Follow-up
1	*Clonorchis sinensis*	N	+/+/+	Albendazole + steroids	15 mth
2	N	*Taenia solium*	+/+/−	Albendazole + Sapylin	12 mth
3	Sparganum^a^	N	+/+/No	Praziquantel + steroids	12 mth
4	N	*Paragonimus westermani*	+/+/No	Praziquantel + Antibiotic	6 mth
5	N	*Paragonimus westermani*	+/−/−	Praziquantel + steroids	10 mth
6	N	*Paragonimus westermani*	+/−/No	Praziquantel + antibiotic	8 mth
7	N	*Paragonimus westermani*	+/+/No	Praziquantel	9 mth
8	N	*Taenia solium*	+/+/+	Praziquantel	9 mth
9	N	*Echinococcus granulosus*	+/+/−	Praziquantel	5 mth
10	N	*Taenia solium*	+/+/No	Praziquantel	5 mth
11	*Clonorchis sinensis*	*Clonorchis sinensis*	+/−/+	Praziquantel	8 mth

^a^ Parasite detected in pleural effusion

Abbreviations*: Eos*, Eosinophils; *mth*, months; *N*, None; *PL*, Pleural effusion; *PPI*, Parasitic pleural infestation

### A practical diagnostic approach for PPI

**Fig. 3:**
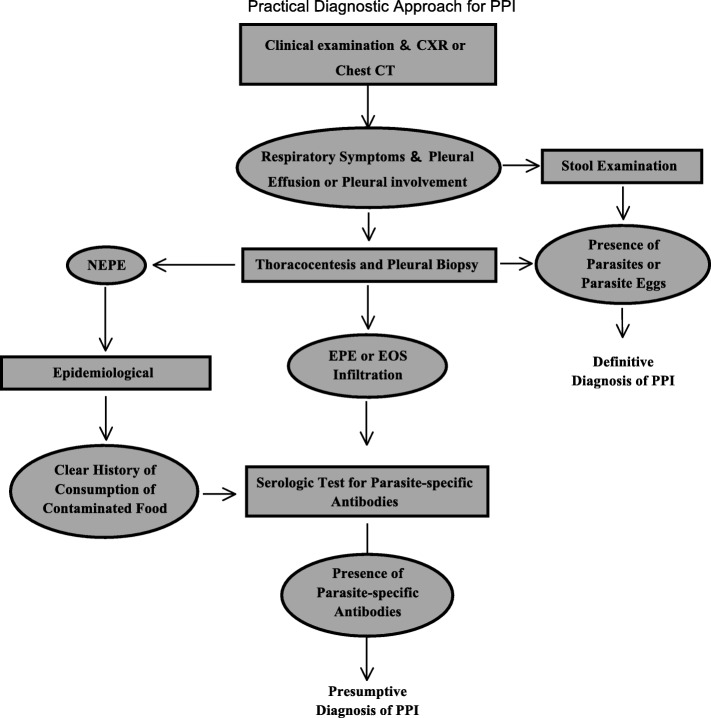
A practical diagnostic approach for the investigation of PPI. The rectangle boxes stated the examination or test procedures. The oval boxes stated the result of upstream examination or test procedures. Abbreviations: PPI, pleural parasitic infestation; CXR, chest X-ray; EPE, eosinophilic pleural effusion; NEPE, non-EPE

The pleural involvement and EPE indicated a possibility of PPIs. The presence of parasites or parasite eggs in stool or pleural effusion supports a definitive diagnosis of PPI. A presumable diagnosis of PPI could be proposed when serum was positive for parasite-specific IgG antibodies. As shown in Fig. 3, a practical diagnostic approach for the PPI is proposed based on the examination and test aforementioned. The algorithm was designed for the diagnosis for Paragonimiasis and may not be applicable in other clinical conditions.

After the diagnosis of PPI was made, all patients were treated with antiparasitic agents. Nine patients were treated with praziquantel (75 mg/kg/day for 2 days) and the other two were treated with albendazole. Antiparasitic treatment resulted in the resolution of symptoms and the abnormal pleural pulmonary radiographic findings in all patients. During follow-up, no recurrences were reported in all the patients.

## Discussion

Data regarding PPI mainly come from a few case reports and small series of patients [3, 11–13]. Mukae et al. and Jeon et al. analyzed the clinical and radiological characteristics associated with pulmonary paragonimiasis in Japanese and Korean patients with or without pleural involvement [3, 12]. In 2015, Hwang et al. retrospectively analyzed the pleural fluid characteristics of pleuropulmonary paragonimiasis [11]. Seon et al. evaluated the chest computed tomography (CT) findings of parasite infestation, finding that pleural effusion was the most common pleural abnormality no matter whether it was caused by paragonimiasis or nonparagonimiatic infestation [13]. However, no satisfactory diagnostic approach or methods have been proposed and the diagnosis of PPI remains a problem in contemporary clinical practice. In the current study, a practical diagnostic approach for PPI was developed after summarizing the clinical characteristics and laboratory data of 11 cases of PPI patients admitted in our institute.

Most patients (9 of 11) included in the current study presented with respiratory symptoms, including shortness of the breath, cough and chest pain. There were 2 patients having fever as the only symptom. The severity of clinical manifestations varied from minor to severe in the 11 PPI patients. Fever (36.4%) and chest pain (27.3%) were more frequently observed in our study than previous reports [3, 13]. The duration of most of the complaints ranged from 2 days to more than 2 years, while fever in these patients lasted less than 1 month. Acute symptoms (such as fever) in these patients may be associated with an acute reaction to parasitic infestation [28]. In the present study, only 5 patients had remarkable intake history of contaminated seafood. Due to its obscure course, little epidemiological evidence, and nonspecific clinical characteristics, these patients were initially misdiagnosed as lung cancer [16] or tuberculosis [11].

Leukocytosis and eosinophilis of peripheral blood can be detected in PPI patients with paragonimiasis [3, 5, 12, 25–28]. In our study, peripheral blood leukocytosis was seen in 45.5% of the patients and eosinophilis was found in 36.4% of the patients, which is similar with the data of previous reports [3, 12, 25–28]. For pleuropulmonary paragonimiasis, eosinophilia is observed only when the worms are alive and migrating and disappears when the disease progresses into its chronic phase [28]. Japanese investigators considered that eosinophilia was an acute-phase response of the host [29]. In our study, eosinophilia was observed in 2 patients for whom the course of the disease tended to be in the chronic phase, suggesting that the absence of eosinophilia in peripheral blood does not necessarily rule out the PPI. Whether other parasites infestation will also lead to the eosinophilia remains unclear [5, 25–27].

Thoracocentesis was carried out to determine the nature of the pleural effusion and to differentiate it from other conditions. The CEA is a representative tumor marker and CEA test is frequently used to define malignant pleural effusion (MPE). Positive result is highly suspicious of MPE, although a normal level of CEA can also be found in malignant condition [30]. In the present study, the values of the CEA in all patients were normal (< 5 mg/mL). Other pleural effusion parameters, such as ADA and LDH, had also been evaluated in previous studies. Patients with paragonimiasis had increased eosinophilia in their pleural effusion which was an exudate with a low level of glucose (< 10 mg/L), a low pH (< 7.10), and a high level of LDH (> 1000 IU/L) [4]. A similar result was found in the study of Jeon et al. [3]. Elevated ADA concentrations (> 40 U/L) were observed in 53% patients in the study of Hwang et al. [11]. These data are not consistent with our findings that showed a relatively low concentration of ADA (11.6 U/L) and LDH 338.2 U/L in the pleural effusion. Therefore, prospective studies with a larger number of patients are required to investigate the actual diagnostic value of some of the pleural effusion parameters.

Interestingly, all patients in our series demonstrated EPE [21], although the degree of eosinophilia varied from patient to patient (10.5–41%). EPEs account for 5 to 16% of exudative pleural effusions and the etiology of EPE includes a great variety of diseases. Oba et al. reported in a meta-analysis that the most common cause of EPEs was malignancy (26%) [22]. Pleural biopsy therefore is required to exclude MPE. It was performed in all the patients in the current study. Eosinophilic infiltration was found in 8 of 11 patients and while there was no evidence of tuberculosis or malignancy in any case. A high incidence in PPI has also been reported in other studies of EPEs, but most of these patients were misdiagnosed [22, 23].

There is a report showing parasite eggs in pleural fluid due to paragonimus infestation [31], but parasite eggs were not seen in any of the pleural biopsies obtained from the current 11 patients. The definitive diagnosis of PPI can be confirmed by the identification of parasites or parasite eggs in pleural effusion or pleural tissue, which is usually challenging because of the low detection rate [29]. Parasite-specific antibody test on serum or other body fluids was also used to support a diagnosis of PPI. However, the value of this test has not been thoroughly evaluated and its importance in the diagnosis of PPI has not been fully appreciated in the clinical practice. In our study, an immunoserologic test for parasite-specific IgG antibodies was performed on serum and pleural effusion, and up to 81.8% patients had positive results. Thus, the presence of parasite-specific antibody seems to be closely related to EPE in PPI. The manifestation of EPE accompanied with a positive test result for parasite-specific antibody strongly suggests the diagnosis of PPI.

In our series, diagnosis of PPI in most EPE patients was made based on the positive data of parasite-specific antibody test. Patients then received anti-parasite treatment which turned out to be effective for the symptom relief and the resolution of pleural effusion. The patient did not show any related symptoms or pleural effusion recurrence under anti-parasitic treatment during follow-up period.

We acknowledge that the present study has a couple of limitations. It was a retrospective study from a single institution and the number of patients evaluated is small due to the low incidence of PPI. Thus, a larger, multicentre, prospective, randomized study is needed for further validation of our results. In addition, while specificity is generally high for Paragonimus serology, false positive results have been detected in patients with other fluke infections such as schistosomiasis.

## Conclusions

In patients with unexplained pleural effusion, parasite-specific IgG antibody tests should be performed when pleural fluid examination shows EPE. Our clinical colleagues should consider a diagnosis of PPI when there is a positive test result for parasite-specific IgG antibodies.

## Data Availability

The datasets supporting the conclusions of this article are included within the article and its figures and tables. Additional data may be available from the corresponding author upon reasonable request.

## Abbreviations

ADA: Adenosine deaminase
CEA: Protein and carcinoembryonic antigen
CT: Computed tomography
ELISA: Enzyme-linked immunosorbent assay
EPE: Eosinophilic pleural effusion
HRCT: High-resolution computed tomography
IEPE: Idiopathic eosinophilic pleural effusion
LDH: Lactate dehydrogenase
MPE: Malignant pleural effusion
PE: Pulmonary embolism
PPE: Parapneumonic pleural effusion
PPI: Pleural parasitic infestation
TPE: Tuberculosis pleural effusion
